# Supplementary material for: Boosting the Electrocatalytic Oxygen Reduction Activity of MnN4-Doped Graphene by Axial Halogen Ligand Modification
Source: Molecules. 2024 Jul 26;29(15):3517. doi: 10.3390/molecules29153517 (PMC11314252; doi:10.3390/molecules29153517)
Supplement: Supplementary file 1 [file molecules-29-03517-s001.zip › molecules-3115038-supplementary.pdf]

Boosting the Electrocatalytic Oxygen Reduction Activity of MnN_4−Doped Graphene by Axial Halogen Ligand Modification

Shaoqiang Wei,[1] Ran Zhao[1], Wenbo Yu[1], Lei Li[1,2,]* and Min Zhang[1,2,]*

[1] *College of Physics and Electronic Information, Inner Mongolia Normal University, Hohhot 010022, China*
[2] *Inner Mongolia Key Laboratory for Physics and Chemistry of Functional Materials, Hohhot 010022, China*

* Corresponding authors.
E-mail address: *lilei@imnu.edu.cn* (L. Li), *zhangm@imnu.edu.cn* (M. Zhang).

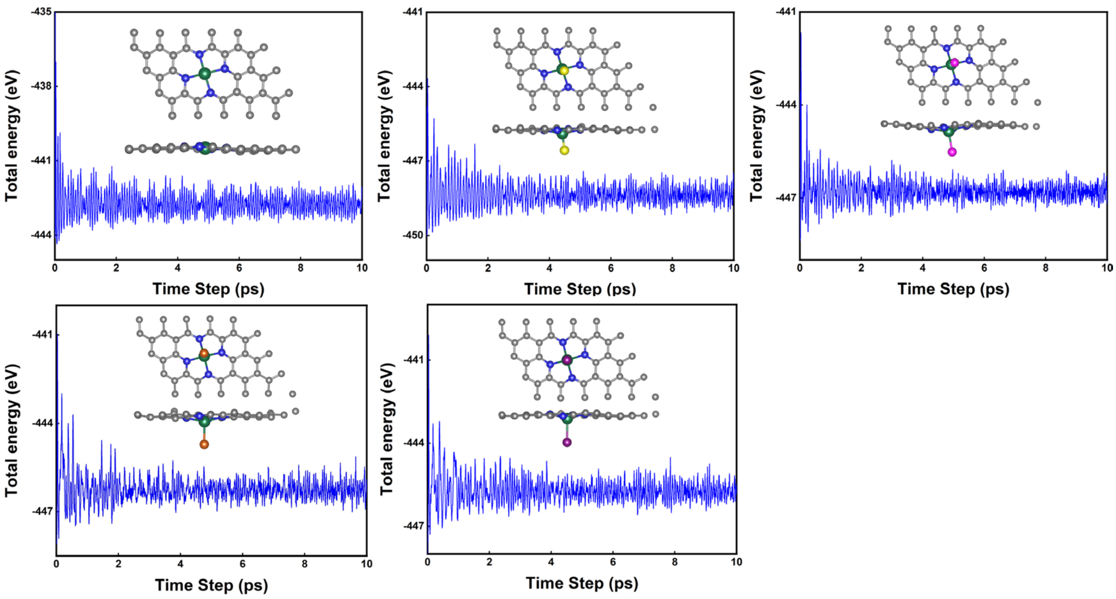

Figure S1. Evolution of energy with the elapsed time for AIMD simulations of MnN_4 and $MnN_4{-}X$ at 300 K. Inserts are top and side views of the snapshot of atomic configuration at 10 ps. Grey, blue, green, yellow, pink, brown and purple balls represent C, N, Mn, F, Cl, Br, and I atoms, respectively.

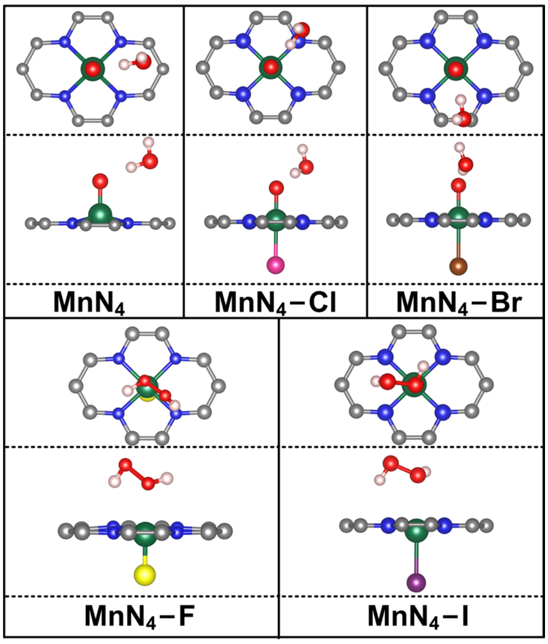

Figure S2. Optimized structures of *HOOH adsorbed on MnN_4 and $MnN_4{-}X$. Red and pink balls represent O and H atoms, respectively.

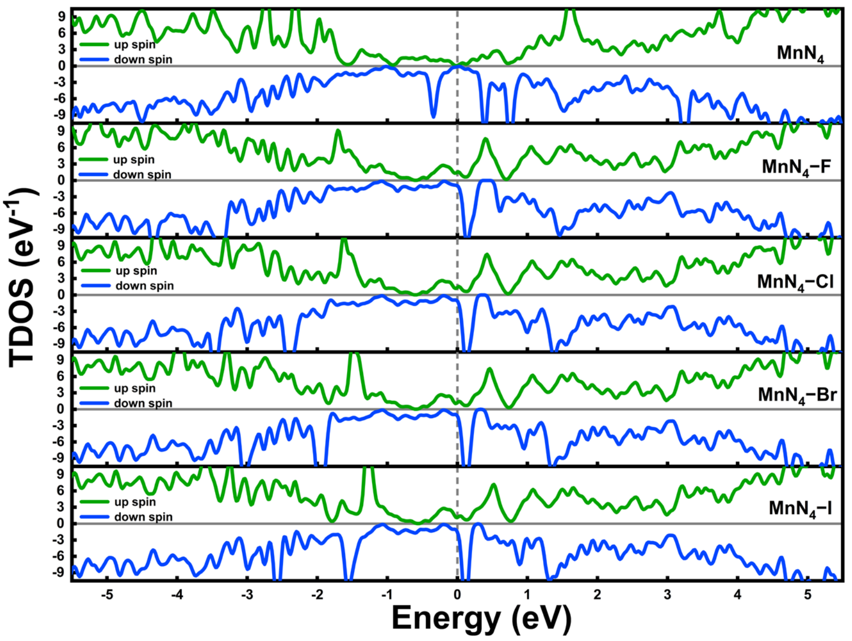

Figure S3. The total density of states (TDOS) of MnN_4 and MnN_4−X.

Table S1. The average Mn−N bond length (d_{Mn-N}), binding energy (E_b), formation energy (E_f), dissolution potential (U_{diss}), and Bader charge transfer for MnN_4 and MnN_4−X. Note that the minus sign in Δq denotes electron loss.

Model	d_{Mn-N} (Å)	E_b (eV)	E_f (eV)	U_{diss} (V)	Δq (e^-)	
					Mn	X (X=F, Cl, Br, and I)
MnN_4	1.916	6.90	−3.16	0.39	−1.28	/
MnN_4−F	1.975	3.82	−6.97	2.30	−1.50	0.65
MnN_4−Cl	1.975	1.72	−4.89	1.25	−1.40	0.59
MnN_4−Br	1.973	1.51	−4.66	1.14	−1.36	0.53
MnN_4−I	1.971	1.17	−4.33	0.97	−1.30	0.47

Table S2. Adsorption energy (E_{ads}) and Gibbs free energy (ΔG) for reaction intermediates.

Model	E_{ads} (eV)				ΔG (eV)			
	$*O_2$	*OOH	*O	*OH	$*O_2$	*OOH	*O	*OH
MnN_4	−1.34	−1.73	−4.89	−2.91	3.50	3.39	0.93	0.34
MnN_4−F	−0.51	−1.50	−3.90	−2.72	4.35	3.61	1.91	0.59
MnN_4−Cl	−0.25	−1.32	−3.84	−2.52	4.54	3.80	1.93	0.79
MnN_4−Br	−0.26	−1.31	−3.85	−2.50	4.52	3.82	1.96	0.82
MnN_4−I	−0.44	−1.32	−3.87	−2.47	4.37	3.81	1.95	0.84

Table S3. Bader charge transfer for intermediates adsorbed MnN_4 and MnN_4–X systems. Note that the minus sign in Δq denotes electron loss.

model	Δq (e^-)											
	*O_2	Mn	X	*OOH	Mn	X	*O	Mn	X	*OH	Mn	X
MnN_4	0.70	−1.48	/	0.47	−1.49	/	0.59	−1.47	/	0.50	−1.51	/
MnN_4–F	0.50	−1.63	0.61	0.48	−1.83	0.61	0.58	−1.58	0.66	0.46	−1.64	0.63
MnN_4–Cl	0.53	−1.53	0.56	0.45	−1.56	0.57	0.56	−1.49	0.59	0.44	−1.54	0.55
MnN_4–Br	0.51	−1.49	0.52	0.50	−1.55	0.54	0.59	−1.51	0.56	0.44	−1.52	0.52
MnN_4–I	0.44	−1.44	0.45	0.50	−1.44	0.46	0.56	−1.43	0.50	0.44	−1.48	0.45
